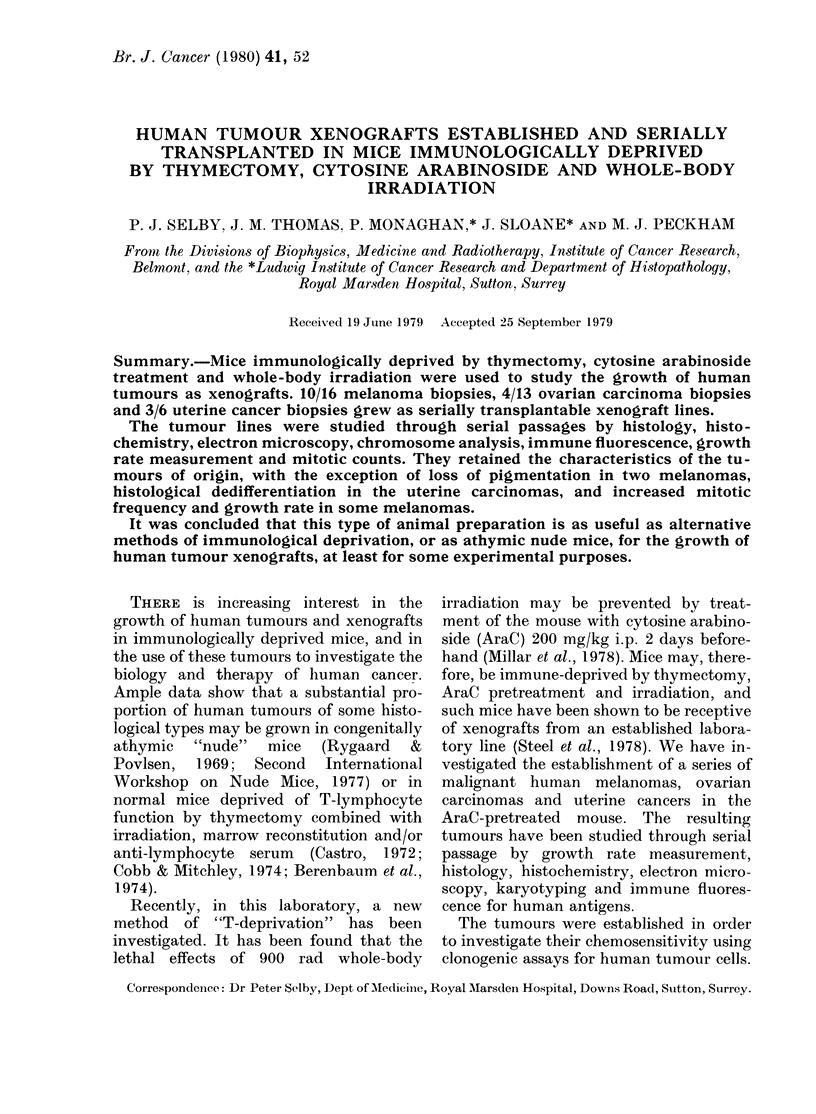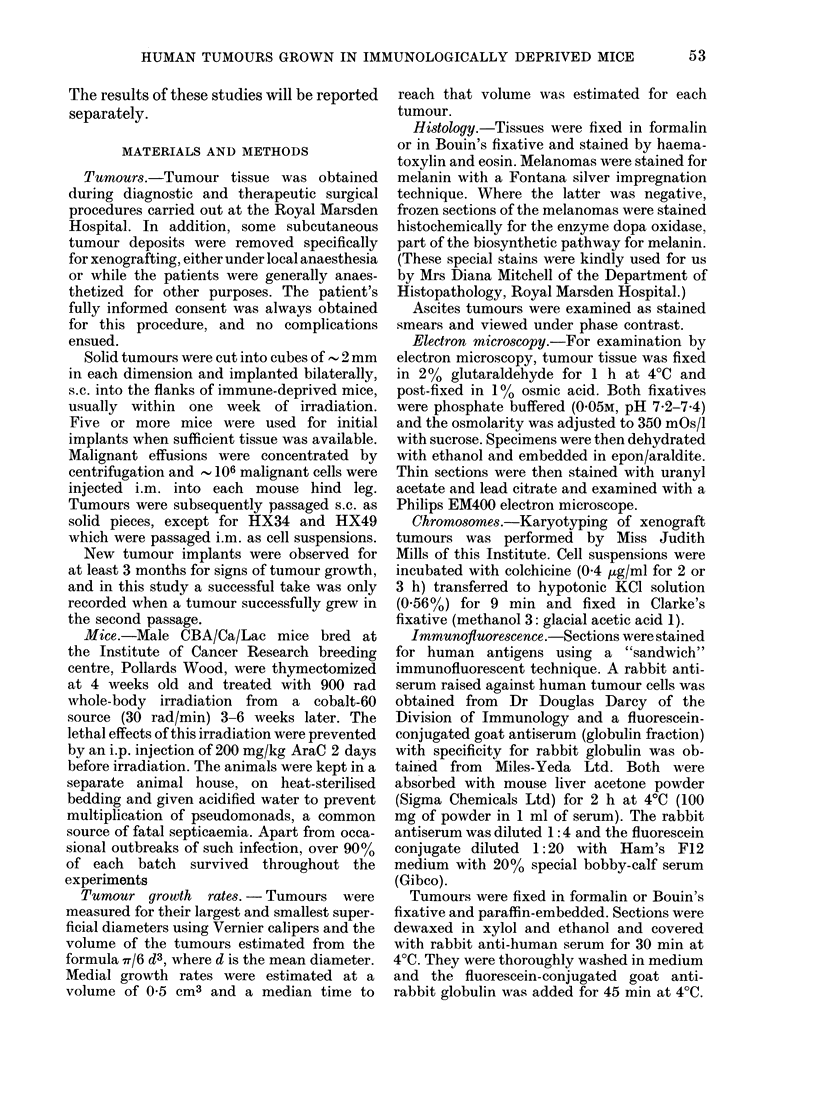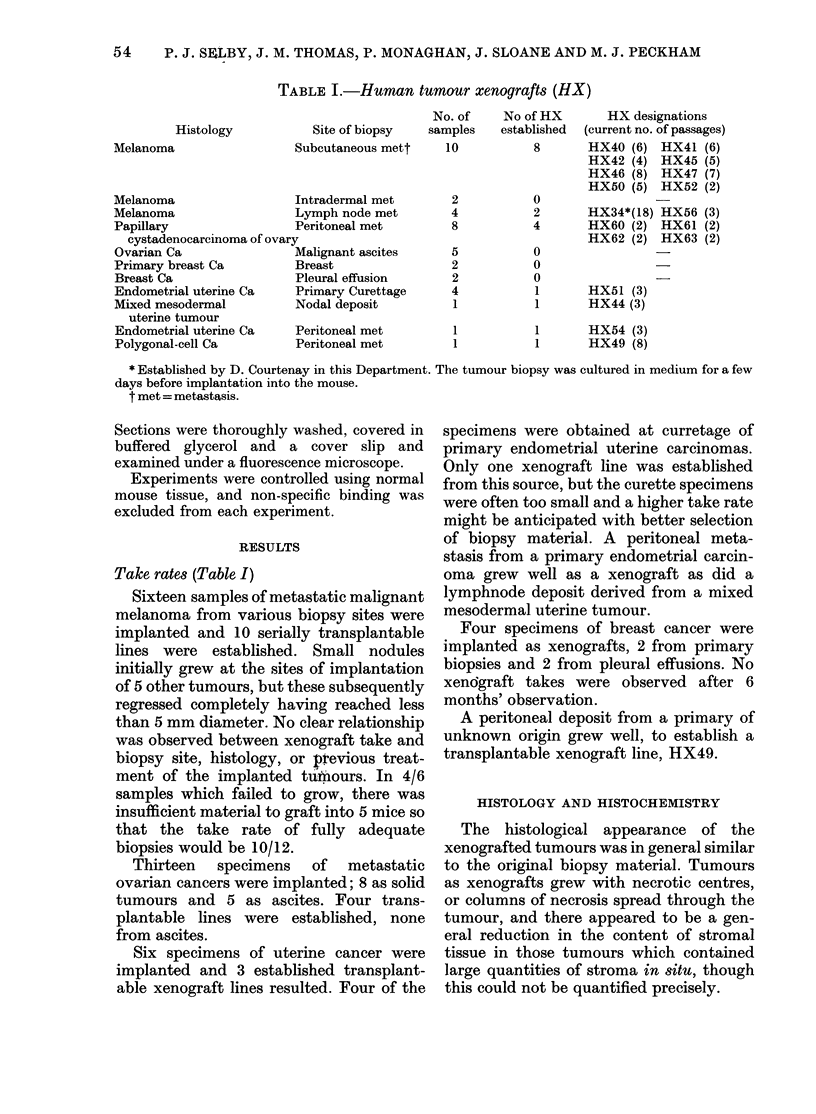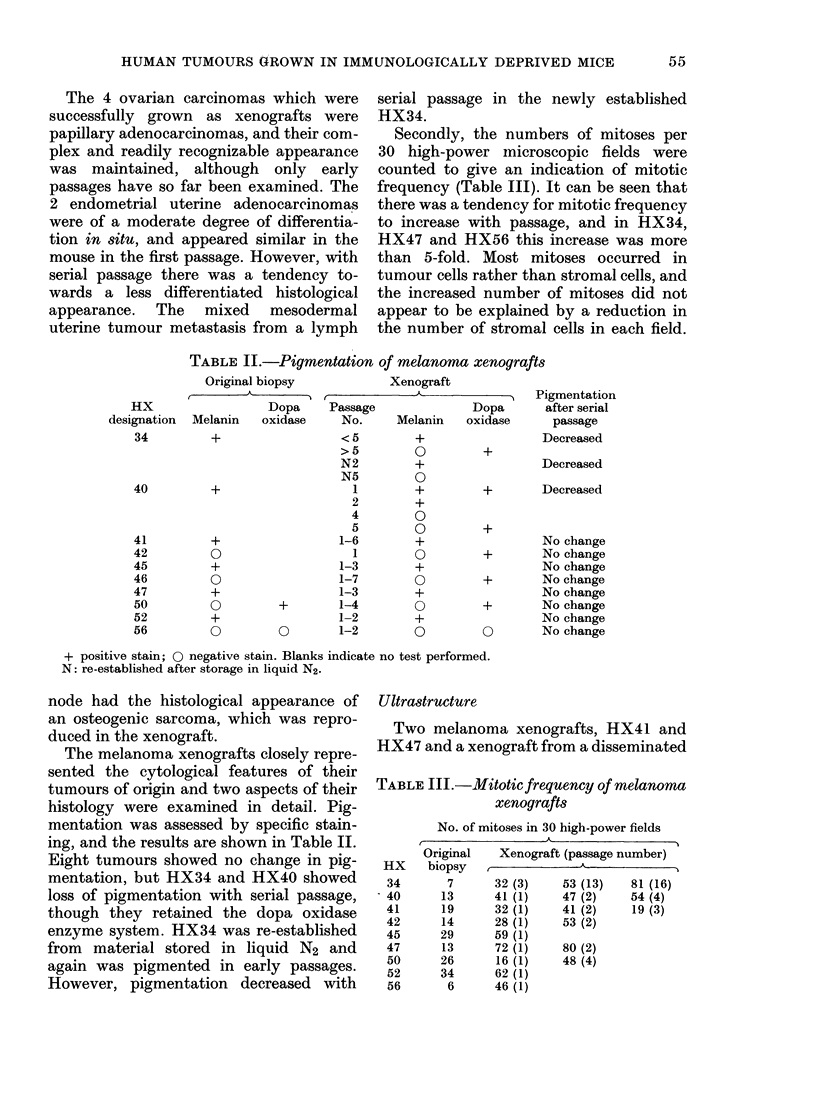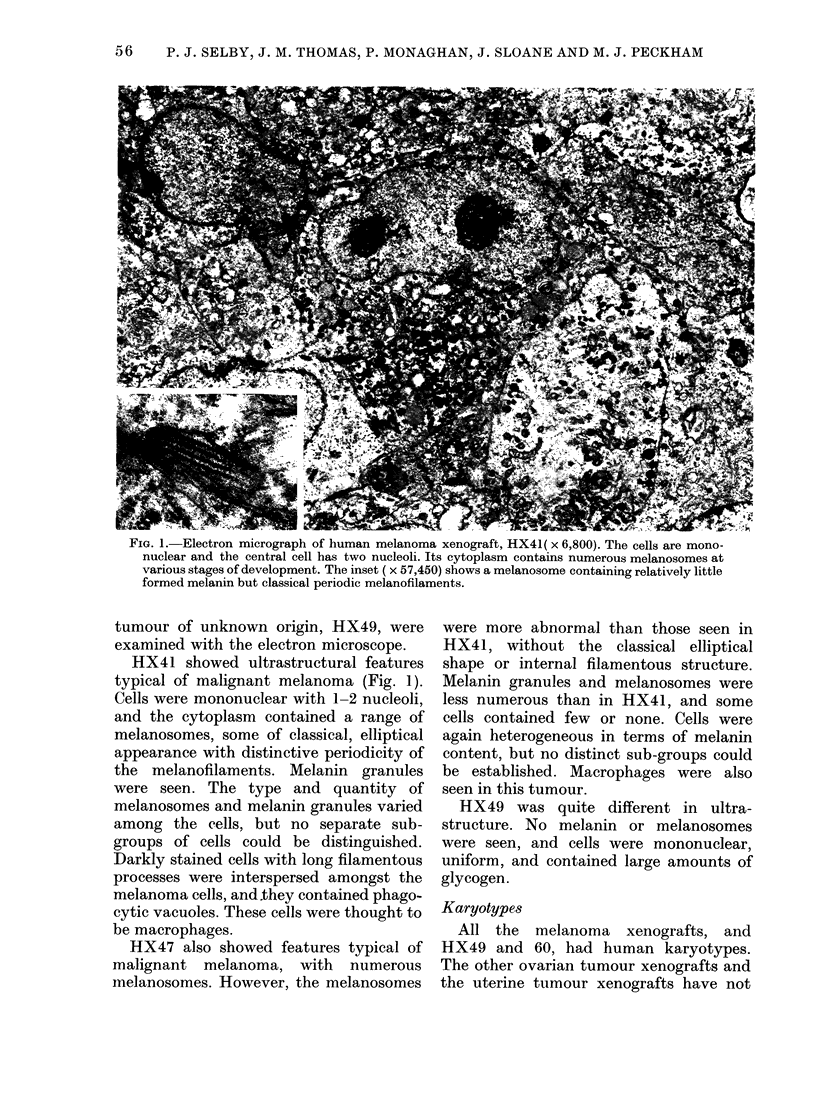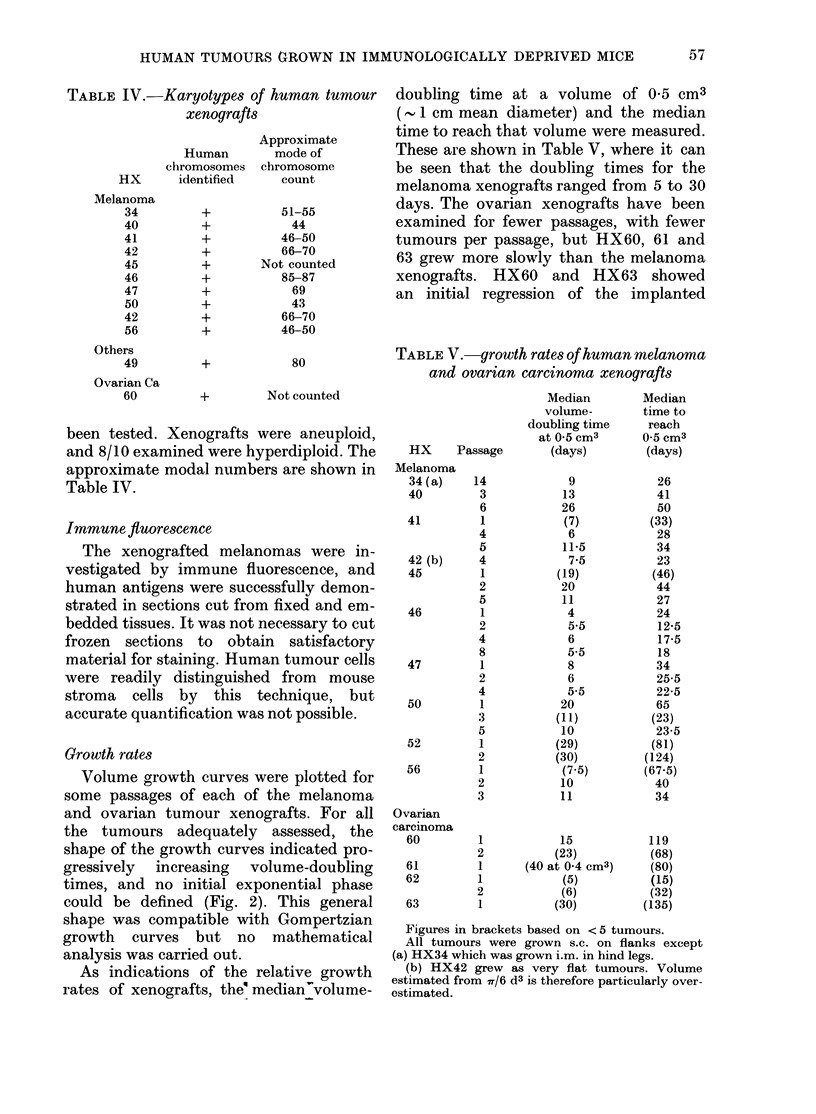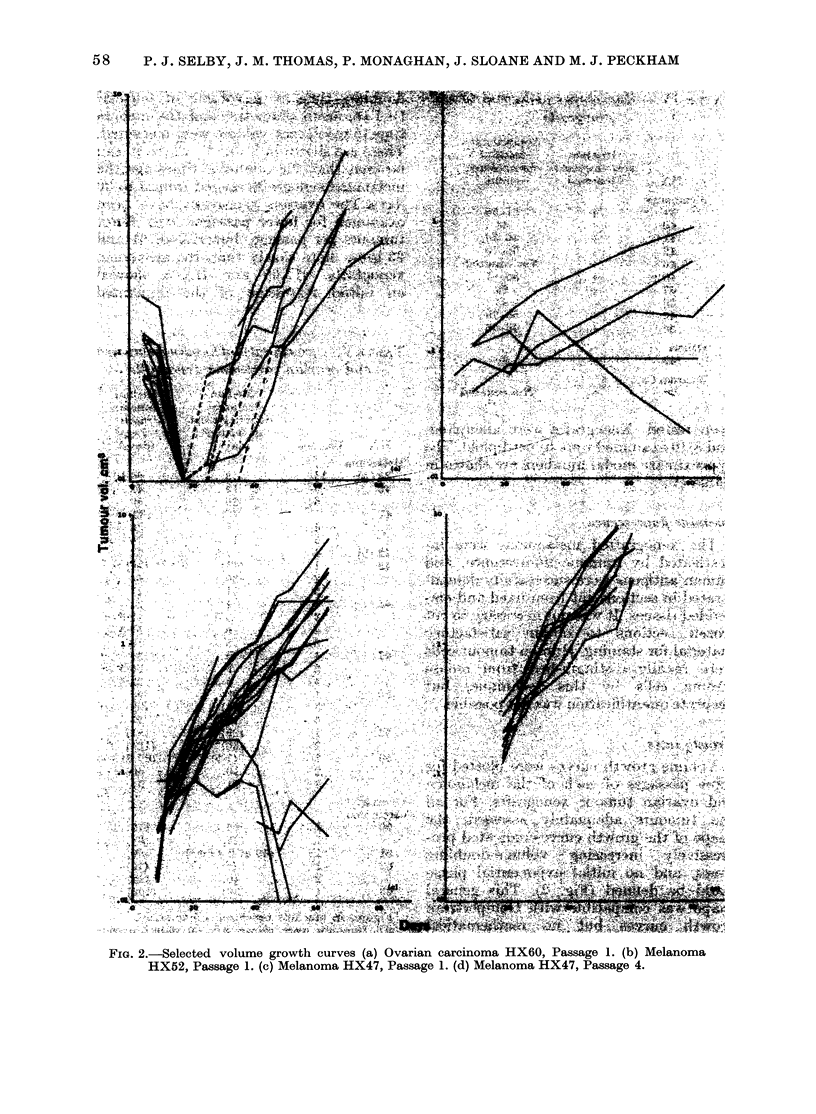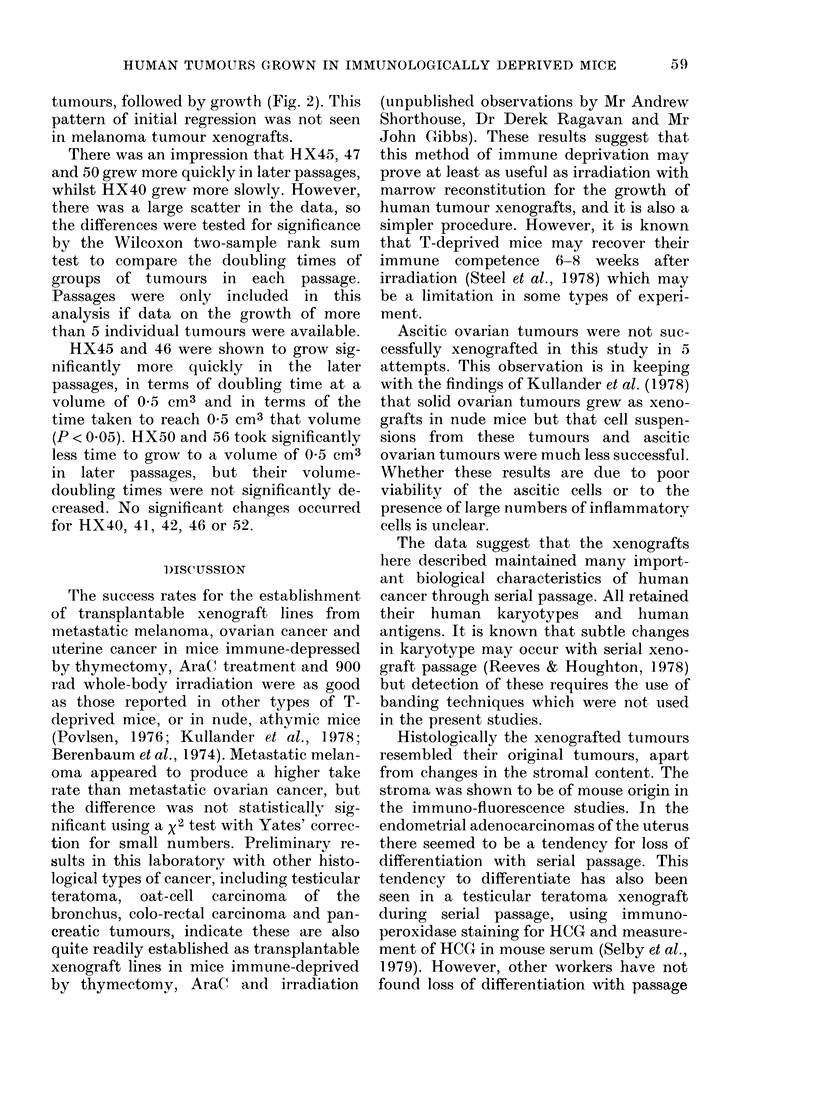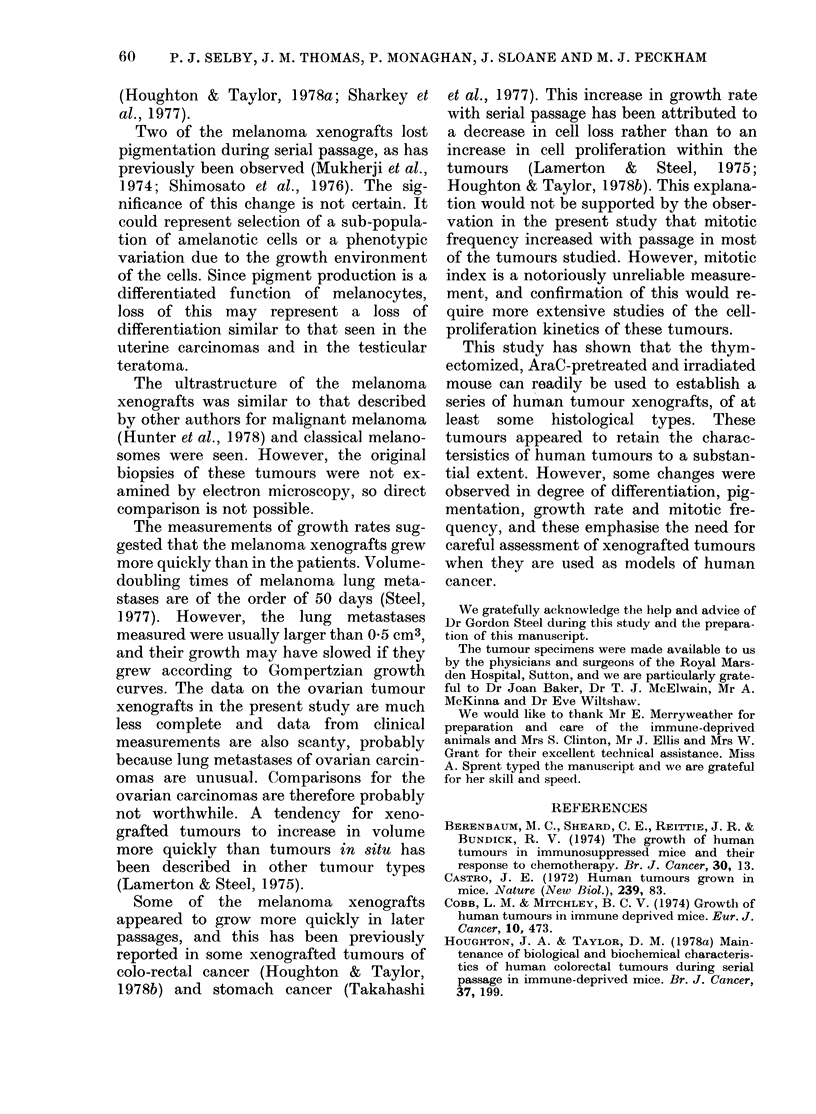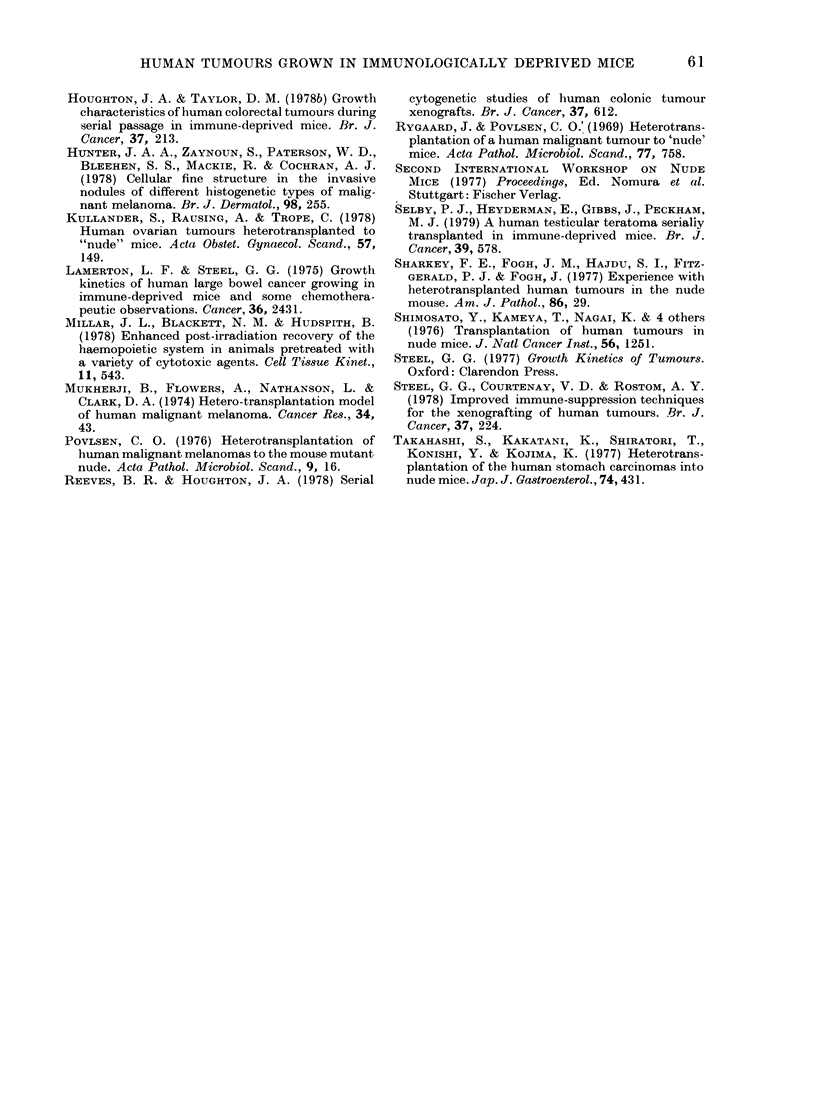# Human tumour xenografts established and serially transplanted in mice immunologically deprived by thymectomy, cytosine arabinoside and whole-body irradiation.

**DOI:** 10.1038/bjc.1980.7

**Published:** 1980-01

**Authors:** P. J. Selby, J. M. Thomas, P. Monaghan, J. Sloane, M. J. Peckham

## Abstract

**Images:**


*Br. J. Cancer* (1980) **41**, 52

### HUMAN TUMOUR XENOGRAFTS ESTABLISHED AND SERIALLY TRANSPLANTED IN MICE IMMUNOLOGICALLY DEPRIVED BY THYMECTOMY, CYTOSINE ARABINOSIDE AND WHOLE-BODY IRRADIATION

P. J. SELBY, J. M. THOMAS, P. MONAGHAN,* J. SLOANE* AND M. J. PECKHAM

*From the Divisions of Biophysics, Medicine and Radiotherapy, Institute of Cancer Research, Belmont, and the *Ludwig Institute of Cancer Research and Department of Histopathology, Royal Marsden Hospital, Sutton, Surrey*

Received 19 June 1979 Accepted 25 September 1979

**Summary.—Mice immunologically deprived by thymectomy, cytosine arabinoside treatment and whole-body irradiation were used to study the growth of human tumours as xenografts. 10/16 melanoma biopsies, 4/13 ovarian carcinoma biopsies and 3/6 uterine cancer biopsies grew as serially transplantable xenograft lines.**

**The tumour lines were studied through serial passages by histology, histochemistry, electron microscopy, chromosome analysis, immune fluorescence, growth rate measurement and mitotic counts. They retained the characteristics of the tumours of origin, with the exception of loss of pigmentation in two melanomas, histological dedifferentiation in the uterine carcinomas, and increased mitotic frequency and growth rate in some melanomas.**

**It was concluded that this type of animal preparation is as useful as alternative methods of immunological deprivation, or as athymic nude mice, for the growth of human tumour xenografts, at least for some experimental purposes.**

THERE is increasing interest in the growth of human tumours and xenografts in immunologically deprived mice, and in the use of these tumours to investigate the biology and therapy of human cancer. Ample data show that a substantial proportion of human tumours of some histological types may be grown in congenitally athymic "nude" mice (Rygaard & Povlsen, 1969; Second International Workshop on Nude Mice, 1977) or in normal mice deprived of T-lymphocyte function by thymectomy combined with irradiation, marrow reconstitution and/or anti-lymphocyte serum (Castro, 1972; Cobb & Mitchley, 1974; Berenbaum *et al.*, 1974).

Recently, in this laboratory, a new method of "T-deprivation" has been investigated. It has been found that the lethal effects of 900 rad whole-body irradiation may be prevented by treatment of the mouse with cytosine arabinoside (AraC) 200 mg/kg i.p. 2 days beforehand (Millar *et al.*, 1978). Mice may, therefore, be immune-deprived by thymectomy, AraC pretreatment and irradiation, and such mice have been shown to be receptive of xenografts from an established laboratory line (Steel *et al.*, 1978). We have investigated the establishment of a series of malignant human melanomas, ovarian carcinomas and uterine cancers in the AraC-pretreated mouse. The resulting tumours have been studied through serial passage by growth rate measurement, histology, histochemistry, electron microscopy, karyotyping and immune fluorescence for human antigens.

The tumours were established in order to investigate their chemosensitivity using clonogenic assays for human tumour cells.

Correspondence: Dr Peter Selby, Dept of Medicine, Royal Marsden Hospital, Downs Road, Sutton, Surrey.

The results of these studies will be reported separately.

### MATERIALS AND METHODS

*Tumours*.—Tumour tissue was obtained during diagnostic and therapeutic surgical procedures carried out at the Royal Marsden Hospital. In addition, some subcutaneous tumour deposits were removed specifically for xenografting, either under local anaesthesia or while the patients were generally anaesthetized for other purposes. The patient's fully informed consent was always obtained for this procedure, and no complications ensued.

Solid tumours were cut into cubes of ~2 mm in each dimension and implanted bilaterally, s.c. into the flanks of immune-deprived mice, usually within one week of irradiation. Five or more mice were used for initial implants when sufficient tissue was available. Malignant effusions were concentrated by centrifugation and $\sim 10^6$ malignant cells were injected i.m. into each mouse hind leg. Tumours were subsequently passaged s.c. as solid pieces, except for HX34 and HX49 which were passaged i.m. as cell suspensions.

New tumour implants were observed for at least 3 months for signs of tumour growth, and in this study a successful take was only recorded when a tumour successfully grew in the second passage.

*Mice*.—Male CBA/Ca/Lac mice bred at the Institute of Cancer Research breeding centre, Pollards Wood, were thymectomized at 4 weeks old and treated with 900 rad whole-body irradiation from a cobalt-60 source (30 rad/min) 3–6 weeks later. The lethal effects of this irradiation were prevented by an i.p. injection of 200 mg/kg AraC 2 days before irradiation. The animals were kept in a separate animal house, on heat-sterilised bedding and given acidified water to prevent multiplication of pseudomonads, a common source of fatal septicaemia. Apart from occasional outbreaks of such infection, over 90% of each batch survived throughout the experiments

*Tumour growth rates*. — Tumours were measured for their largest and smallest superficial diameters using Vernier calipers and the volume of the tumours estimated from the formula $\pi/6\ d^3$, where $d$ is the mean diameter. Medial growth rates were estimated at a volume of 0·5 $cm^3$ and a median time to reach that volume was estimated for each tumour.

*Histology*.—Tissues were fixed in formalin or in Bouin's fixative and stained by haematoxylin and eosin. Melanomas were stained for melanin with a Fontana silver impregnation technique. Where the latter was negative, frozen sections of the melanomas were stained histochemically for the enzyme dopa oxidase, part of the biosynthetic pathway for melanin. (These special stains were kindly used for us by Mrs Diana Mitchell of the Department of Histopathology, Royal Marsden Hospital.)

Ascites tumours were examined as stained smears and viewed under phase contrast.

*Electron microscopy*.—For examination by electron microscopy, tumour tissue was fixed in 2% glutaraldehyde for 1 h at 4°C and post-fixed in 1% osmic acid. Both fixatives were phosphate buffered (0·05M, pH 7·2–7·4) and the osmolarity was adjusted to 350 mOs/l with sucrose. Specimens were then dehydrated with ethanol and embedded in epon/araldite. Thin sections were then stained with uranyl acetate and lead citrate and examined with a Philips EM400 electron microscope.

*Chromosomes*.—Karyotyping of xenograft tumours was performed by Miss Judith Mills of this Institute. Cell suspensions were incubated with colchicine (0·4 μg/ml for 2 or 3 h) transferred to hypotonic KCl solution (0·56%) for 9 min and fixed in Clarke's fixative (methanol 3: glacial acetic acid 1).

*Immunofluorescence*.—Sections were stained for human antigens using a "sandwich" immunofluorescent technique. A rabbit antiserum raised against human tumour cells was obtained from Dr Douglas Darcy of the Division of Immunology and a fluorescein-conjugated goat antiserum (globulin fraction) with specificity for rabbit globulin was obtained from Miles-Yeda Ltd. Both were absorbed with mouse liver acetone powder (Sigma Chemicals Ltd) for 2 h at 4°C (100 mg of powder in 1 ml of serum). The rabbit antiserum was diluted 1:4 and the fluorescein conjugate diluted 1:20 with Ham's F12 medium with 20% special bobby-calf serum (Gibco).

Tumours were fixed in formalin or Bouin's fixative and paraffin-embedded. Sections were dewaxed in xylol and ethanol and covered with rabbit anti-human serum for 30 min at 4°C. They were thoroughly washed in medium and the fluorescein-conjugated goat anti-rabbit globulin was added for 45 min at 4°C.

TABLE I.—*Human tumour xenografts* (*HX*)

| Histology | Site of biopsy | No. of samples | No of HX established | HX designations (current no. of passages) |
|---|---|---|---|---|
| Melanoma | Subcutaneous met† | 10 | 8 | HX40 (6) HX41 (6) HX42 (4) HX45 (5) HX46 (8) HX47 (7) HX50 (5) HX52 (2) |
| Melanoma | Intradermal met | 2 | 0 | — |
| Melanoma | Lymph node met | 4 | 2 | HX34*(18) HX56 (3) |
| Papillary cystadenocarcinoma of ovary | Peritoneal met | 8 | 4 | HX60 (2) HX61 (2) HX62 (2) HX63 (2) |
| Ovarian Ca | Malignant ascites | 5 | 0 | — |
| Primary breast Ca | Breast | 2 | 0 | — |
| Breast Ca | Pleural effusion | 2 | 0 | — |
| Endometrial uterine Ca | Primary Curettage | 4 | 1 | HX51 (3) |
| Mixed mesodermal uterine tumour | Nodal deposit | 1 | 1 | HX44 (3) |
| Endometrial uterine Ca | Peritoneal met | 1 | 1 | HX54 (3) |
| Polygonal-cell Ca | Peritoneal met | 1 | 1 | HX49 (8) |

* Established by D. Courtenay in this Department. The tumour biopsy was cultured in medium for a few days before implantation into the mouse.
† met = metastasis.

Sections were thoroughly washed, covered in buffered glycerol and a cover slip and examined under a fluorescence microscope.

Experiments were controlled using normal mouse tissue, and non-specific binding was excluded from each experiment.

### RESULTS

### *Take rates* (*Table I*)

Sixteen samples of metastatic malignant melanoma from various biopsy sites were implanted and 10 serially transplantable lines were established. Small nodules initially grew at the sites of implantation of 5 other tumours, but these subsequently regressed completely having reached less than 5 mm diameter. No clear relationship was observed between xenograft take and biopsy site, histology, or previous treatment of the implanted tumours. In 4/6 samples which failed to grow, there was insufficient material to graft into 5 mice so that the take rate of fully adequate biopsies would be 10/12.

Thirteen specimens of metastatic ovarian cancers were implanted; 8 as solid tumours and 5 as ascites. Four transplantable lines were established, none from ascites.

Six specimens of uterine cancer were implanted and 3 established transplantable xenograft lines resulted. Four of the specimens were obtained at curretage of primary endometrial uterine carcinomas. Only one xenograft line was established from this source, but the curette specimens were often too small and a higher take rate might be anticipated with better selection of biopsy material. A peritoneal metastasis from a primary endometrial carcinoma grew well as a xenograft as did a lymphnode deposit derived from a mixed mesodermal uterine tumour.

Four specimens of breast cancer were implanted as xenografts, 2 from primary biopsies and 2 from pleural effusions. No xenograft takes were observed after 6 months' observation.

A peritoneal deposit from a primary of unknown origin grew well, to establish a transplantable xenograft line, HX49.

### HISTOLOGY AND HISTOCHEMISTRY

The histological appearance of the xenografted tumours was in general similar to the original biopsy material. Tumours as xenografts grew with necrotic centres, or columns of necrosis spread through the tumour, and there appeared to be a general reduction in the content of stromal tissue in those tumours which contained large quantities of stroma *in situ*, though this could not be quantified precisely.

The 4 ovarian carcinomas which were successfully grown as xenografts were papillary adenocarcinomas, and their complex and readily recognizable appearance was maintained, although only early passages have so far been examined. The 2 endometrial uterine adenocarcinomas were of a moderate degree of differentiation *in situ*, and appeared similar in the mouse in the first passage. However, with serial passage there was a tendency towards a less differentiated histological appearance. The mixed mesodermal uterine tumour metastasis from a lymph node had the histological appearance of an osteogenic sarcoma, which was reproduced in the xenograft.

The melanoma xenografts closely represented the cytological features of their tumours of origin and two aspects of their histology were examined in detail. Pigmentation was assessed by specific staining, and the results are shown in Table II. Eight tumours showed no change in pigmentation, but HX34 and HX40 showed loss of pigmentation with serial passage, though they retained the dopa oxidase enzyme system. HX34 was re-established from material stored in liquid $N_2$ and again was pigmented in early passages. However, pigmentation decreased with serial passage in the newly established HX34.

Secondly, the numbers of mitoses per 30 high-power microscopic fields were counted to give an indication of mitotic frequency (Table III). It can be seen that there was a tendency for mitotic frequency to increase with passage, and in HX34, HX47 and HX56 this increase was more than 5-fold. Most mitoses occurred in tumour cells rather than stromal cells, and the increased number of mitoses did not appear to be explained by a reduction in the number of stromal cells in each field.

TABLE II.—*Pigmentation of melanoma xenografts*

| | Original biopsy | | Xenograft | | | |
|---|---|---|---|---|---|---|
| HX designation | Melanin | Dopa oxidase | Passage No. | Melanin | Dopa oxidase | Pigmentation after serial passage |
| 34 | + | | < 5 | + | | Decreased |
| | | | > 5 | ○ | + | |
| | | | N2 | + | | Decreased |
| | | | N5 | ○ | | |
| 40 | + | | 1 | + | + | Decreased |
| | | | 2 | + | | |
| | | | 4 | ○ | | |
| | | | 5 | ○ | + | |
| 41 | + | | 1–6 | + | | No change |
| 42 | ○ | | 1 | ○ | + | No change |
| 45 | + | | 1–3 | + | | No change |
| 46 | ○ | | 1–7 | ○ | + | No change |
| 47 | + | | 1–3 | + | | No change |
| 50 | ○ | + | 1–4 | ○ | + | No change |
| 52 | + | | 1–2 | + | | No change |
| 56 | ○ | ○ | 1–2 | ○ | ○ | No change |

+ positive stain; ○ negative stain. Blanks indicate no test performed.
N: re-established after storage in liquid $N_2$.

*Ultrastructure*

Two melanoma xenografts, HX41 and HX47 and a xenograft from a disseminated

TABLE III.—*Mitotic frequency of melanoma xenografts*

| | No. of mitoses in 30 high-power fields | | | |
|---|---|---|---|---|
| HX | Original biopsy | Xenograft (passage number) | | |
| 34 | 7 | 32 (3) | 53 (13) | 81 (16) |
| 40 | 13 | 41 (1) | 47 (2) | 54 (4) |
| 41 | 19 | 32 (1) | 41 (2) | 19 (3) |
| 42 | 14 | 28 (1) | 53 (2) | |
| 45 | 29 | 59 (1) | | |
| 47 | 13 | 72 (1) | 80 (2) | |
| 50 | 26 | 16 (1) | 48 (4) | |
| 52 | 34 | 62 (1) | | |
| 56 | 6 | 46 (1) | | |

FIG. 1.—Electron micrograph of human melanoma xenograft, HX41( × 6,800). The cells are mononuclear and the central cell has two nucleoli. Its cytoplasm contains numerous melanosomes at various stages of development. The inset ( × 57,450) shows a melanosome containing relatively little formed melanin but classical periodic melanofilaments.

tumour of unknown origin, HX49, were examined with the electron microscope.

HX41 showed ultrastructural features typical of malignant melanoma (Fig. 1). Cells were mononuclear with 1–2 nucleoli, and the cytoplasm contained a range of melanosomes, some of classical, elliptical appearance with distinctive periodicity of the melanofilaments. Melanin granules were seen. The type and quantity of melanosomes and melanin granules varied among the cells, but no separate subgroups of cells could be distinguished. Darkly stained cells with long filamentous processes were interspersed amongst the melanoma cells, and they contained phagocytic vacuoles. These cells were thought to be macrophages.

HX47 also showed features typical of malignant melanoma, with numerous melanosomes. However, the melanosomes were more abnormal than those seen in HX41, without the classical elliptical shape or internal filamentous structure. Melanin granules and melanosomes were less numerous than in HX41, and some cells contained few or none. Cells were again heterogeneous in terms of melanin content, but no distinct sub-groups could be established. Macrophages were also seen in this tumour.

HX49 was quite different in ultrastructure. No melanin or melanosomes were seen, and cells were mononuclear, uniform, and contained large amounts of glycogen.

*Karyotypes*

All the melanoma xenografts, and HX49 and 60, had human karyotypes. The other ovarian tumour xenografts and the uterine tumour xenografts have not

TABLE IV.—*Karyotypes of human tumour xenografts*

| HX | Human chromosomes identified | Approximate mode of chromosome count |
|---|---|---|
| Melanoma | | |
| 34 | + | 51–55 |
| 40 | + | 44 |
| 41 | + | 46–50 |
| 42 | + | 66–70 |
| 45 | + | Not counted |
| 46 | + | 85–87 |
| 47 | + | 69 |
| 50 | + | 43 |
| 42 | + | 66–70 |
| 56 | + | 46–50 |
| Others | | |
| 49 | + | 80 |
| Ovarian Ca | | |
| 60 | + | Not counted |

been tested. Xenografts were aneuploid, and 8/10 examined were hyperdiploid. The approximate modal numbers are shown in Table IV.

*Immune fluorescence*

The xenografted melanomas were investigated by immune fluorescence, and human antigens were successfully demonstrated in sections cut from fixed and embedded tissues. It was not necessary to cut frozen sections to obtain satisfactory material for staining. Human tumour cells were readily distinguished from mouse stroma cells by this technique, but accurate quantification was not possible.

*Growth rates*

Volume growth curves were plotted for some passages of each of the melanoma and ovarian tumour xenografts. For all the tumours adequately assessed, the shape of the growth curves indicated progressively increasing volume-doubling times, and no initial exponential phase could be defined (Fig. 2). This general shape was compatible with Gompertzian growth curves but no mathematical analysis was carried out.

As indications of the relative growth rates of xenografts, the median volume-doubling time at a volume of 0·5 $cm^3$ ($\sim$1 cm mean diameter) and the median time to reach that volume were measured. These are shown in Table V, where it can be seen that the doubling times for the melanoma xenografts ranged from 5 to 30 days. The ovarian xenografts have been examined for fewer passages, with fewer tumours per passage, but HX60, 61 and 63 grew more slowly than the melanoma xenografts. HX60 and HX63 showed an initial regression of the implanted

TABLE V.—*growth rates of human melanoma and ovarian carcinoma xenografts*

| HX | Passage | Median volume-doubling time at 0·5 $cm^3$ (days) | Median time to reach 0·5 $cm^3$ (days) |
|---|---|---|---|
| Melanoma | | | |
| 34 (a) | 14 | 9 | 26 |
| 40 | 3 | 13 | 41 |
| | 6 | 26 | 50 |
| 41 | 1 | (7) | (33) |
| | 4 | 6 | 28 |
| | 5 | 11·5 | 34 |
| 42 (b) | 4 | 7·5 | 23 |
| 45 | 1 | (19) | (46) |
| | 2 | 20 | 44 |
| | 5 | 11 | 27 |
| 46 | 1 | 4 | 24 |
| | 2 | 5·5 | 12·5 |
| | 4 | 6 | 17·5 |
| | 8 | 5·5 | 18 |
| 47 | 1 | 8 | 34 |
| | 2 | 6 | 25·5 |
| | 4 | 5·5 | 22·5 |
| 50 | 1 | 20 | 65 |
| | 3 | (11) | (23) |
| | 5 | 10 | 23·5 |
| 52 | 1 | (29) | (81) |
| | 2 | (30) | (124) |
| 56 | 1 | (7·5) | (67·5) |
| | 2 | 10 | 40 |
| | 3 | 11 | 34 |
| Ovarian carcinoma | | | |
| 60 | 1 | 15 | 119 |
| | 2 | (23) | (68) |
| 61 | 1 | (40 at 0·4 $cm^3$) | (80) |
| 62 | 1 | (5) | (15) |
| | 2 | (6) | (32) |
| 63 | 1 | (30) | (135) |

Figures in brackets based on < 5 tumours.
All tumours were grown s.c. on flanks except (a) HX34 which was grown i.m. in hind legs.
(b) HX42 grew as very flat tumours. Volume estimated from $\pi/6\ d^3$ is therefore particularly overestimated.

FIG. 2.—Selected volume growth curves (a) Ovarian carcinoma HX60, Passage 1. (b) Melanoma HX52, Passage 1. (c) Melanoma HX47, Passage 1. (d) Melanoma HX47, Passage 4.

tumours, followed by growth (Fig. 2). This pattern of initial regression was not seen in melanoma tumour xenografts.

There was an impression that HX45, 47 and 50 grew more quickly in later passages, whilst HX40 grew more slowly. However, there was a large scatter in the data, so the differences were tested for significance by the Wilcoxon two-sample rank sum test to compare the doubling times of groups of tumours in each passage. Passages were only included in this analysis if data on the growth of more than 5 individual tumours were available.

HX45 and 46 were shown to grow significantly more quickly in the later passages, in terms of doubling time at a volume of 0·5 $cm^3$ and in terms of the time taken to reach 0·5 $cm^3$ that volume ($P < 0{\cdot}05$). HX50 and 56 took significantly less time to grow to a volume of 0·5 $cm^3$ in later passages, but their volume-doubling times were not significantly decreased. No significant changes occurred for HX40, 41, 42, 46 or 52.

### DISCUSSION

The success rates for the establishment of transplantable xenograft lines from metastatic melanoma, ovarian cancer and uterine cancer in mice immune-depressed by thymectomy, AraC treatment and 900 rad whole-body irradiation were as good as those reported in other types of T-deprived mice, or in nude, athymic mice (Povlsen, 1976; Kullander *et al.*, 1978; Berenbaum *et al.*, 1974). Metastatic melanoma appeared to produce a higher take rate than metastatic ovarian cancer, but the difference was not statistically significant using a $\chi^2$ test with Yates' correction for small numbers. Preliminary results in this laboratory with other histological types of cancer, including testicular teratoma, oat-cell carcinoma of the bronchus, colo-rectal carcinoma and pancreatic tumours, indicate these are also quite readily established as transplantable xenograft lines in mice immune-deprived by thymectomy, AraC and irradiation (unpublished observations by Mr Andrew Shorthouse, Dr Derek Ragavan and Mr John Gibbs). These results suggest that this method of immune deprivation may prove at least as useful as irradiation with marrow reconstitution for the growth of human tumour xenografts, and it is also a simpler procedure. However, it is known that T-deprived mice may recover their immune competence 6–8 weeks after irradiation (Steel *et al.*, 1978) which may be a limitation in some types of experiment.

Ascitic ovarian tumours were not successfully xenografted in this study in 5 attempts. This observation is in keeping with the findings of Kullander *et al.* (1978) that solid ovarian tumours grew as xenografts in nude mice but that cell suspensions from these tumours and ascitic ovarian tumours were much less successful. Whether these results are due to poor viability of the ascitic cells or to the presence of large numbers of inflammatory cells is unclear.

The data suggest that the xenografts here described maintained many important biological characteristics of human cancer through serial passage. All retained their human karyotypes and human antigens. It is known that subtle changes in karyotype may occur with serial xenograft passage (Reeves & Houghton, 1978) but detection of these requires the use of banding techniques which were not used in the present studies.

Histologically the xenografted tumours resembled their original tumours, apart from changes in the stromal content. The stroma was shown to be of mouse origin in the immuno-fluorescence studies. In the endometrial adenocarcinomas of the uterus there seemed to be a tendency for loss of differentiation with serial passage. This tendency to differentiate has also been seen in a testicular teratoma xenograft during serial passage, using immunoperoxidase staining for HCG and measurement of HCG in mouse serum (Selby *et al.*, 1979). However, other workers have not found loss of differentiation with passage

(Houghton & Taylor, 1978*a*; Sharkey *et al.*, 1977).

Two of the melanoma xenografts lost pigmentation during serial passage, as has previously been observed (Mukherji *et al.*, 1974; Shimosato *et al.*, 1976). The significance of this change is not certain. It could represent selection of a sub-population of amelanotic cells or a phenotypic variation due to the growth environment of the cells. Since pigment production is a differentiated function of melanocytes, loss of this may represent a loss of differentiation similar to that seen in the uterine carcinomas and in the testicular teratoma.

The ultrastructure of the melanoma xenografts was similar to that described by other authors for malignant melanoma (Hunter *et al.*, 1978) and classical melanosomes were seen. However, the original biopsies of these tumours were not examined by electron microscopy, so direct comparison is not possible.

The measurements of growth rates suggested that the melanoma xenografts grew more quickly than in the patients. Volume-doubling times of melanoma lung metastases are of the order of 50 days (Steel, 1977). However, the lung metastases measured were usually larger than 0·5 cm$^3$, and their growth may have slowed if they grew according to Gompertzian growth curves. The data on the ovarian tumour xenografts in the present study are much less complete and data from clinical measurements are also scanty, probably because lung metastases of ovarian carcinomas are unusual. Comparisons for the ovarian carcinomas are therefore probably not worthwhile. A tendency for xenografted tumours to increase in volume more quickly than tumours *in situ* has been described in other tumour types (Lamerton & Steel, 1975).

Some of the melanoma xenografts appeared to grow more quickly in later passages, and this has been previously reported in some xenografted tumours of colo-rectal cancer (Houghton & Taylor, 1978*b*) and stomach cancer (Takahashi *et al.*, 1977). This increase in growth rate with serial passage has been attributed to a decrease in cell loss rather than to an increase in cell proliferation within the tumours (Lamerton & Steel, 1975; Houghton & Taylor, 1978*b*). This explanation would not be supported by the observation in the present study that mitotic frequency increased with passage in most of the tumours studied. However, mitotic index is a notoriously unreliable measurement, and confirmation of this would require more extensive studies of the cell-proliferation kinetics of these tumours.

This study has shown that the thymectomized, AraC-pretreated and irradiated mouse can readily be used to establish a series of human tumour xenografts, of at least some histological types. These tumours appeared to retain the charactersistics of human tumours to a substantial extent. However, some changes were observed in degree of differentiation, pigmentation, growth rate and mitotic frequency, and these emphasise the need for careful assessment of xenografted tumours when they are used as models of human cancer.

We gratefully acknowledge the help and advice of Dr Gordon Steel during this study and the preparation of this manuscript.

The tumour specimens were made available to us by the physicians and surgeons of the Royal Marsden Hospital, Sutton, and we are particularly grateful to Dr Joan Baker, Dr T. J. McElwain, Mr A. McKinna and Dr Eve Wiltshaw.

We would like to thank Mr E. Merryweather for preparation and care of the immune-deprived animals and Mrs S. Clinton, Mr J. Ellis and Mrs W. Grant for their excellent technical assistance. Miss A. Sprent typed the manuscript and we are grateful for her skill and speed.

### REFERENCES

Berenbaum, M. C., Sheard, C. E., Reittie, J. R. & Bundick, R. V. (1974) The growth of human tumours in immunosuppressed mice and their response to chemotherapy. *Br. J. Cancer*, **30**, 13.

Castro, J. E. (1972) Human tumours grown in mice. *Nature (New Biol.)*, **239**, 83.

Cobb, L. M. & Mitchley, B. C. V. (1974) Growth of human tumours in immune deprived mice. *Eur. J. Cancer*, **10**, 473.

Houghton, J. A. & Taylor, D. M. (1978*a*) Maintenance of biological and biochemical characteristics of human colorectal tumours during serial passage in immune-deprived mice. *Br. J. Cancer*, **37**, 199.

HOUGHTON, J. A. & TAYLOR, D. M. (1978b) Growth characteristics of human colorectal tumours during serial passage in immune-deprived mice. *Br. J. Cancer*, **37**, 213.

HUNTER, J. A. A., ZAYNOUN, S., PATERSON, W. D., BLEEHEN, S. S., MACKIE, R. & COCHRAN, A. J. (1978) Cellular fine structure in the invasive nodules of different histogenetic types of malignant melanoma. *Br. J. Dermatol.*, **98**, 255.

KULLANDER, S., RAUSING, A. & TROPE, C. (1978) Human ovarian tumours heterotransplanted to "nude" mice. *Acta Obstet. Gynaecol. Scand.*, **57**, 149.

LAMERTON, L. F. & STEEL, G. G. (1975) Growth kinetics of human large bowel cancer growing in immune-deprived mice and some chemotherapeutic observations. *Cancer*, **36**, 2431.

MILLAR, J. L., BLACKETT, N. M. & HUDSPITH, B. (1978) Enhanced post-irradiation recovery of the haemopoietic system in animals pretreated with a variety of cytotoxic agents. *Cell Tissue Kinet.*, **11**, 543.

MUKHERJI, B., FLOWERS, A., NATHANSON, L. & CLARK, D. A. (1974) Hetero-transplantation model of human malignant melanoma. *Cancer Res.*, **34**, 43.

POVLSEN, C. O. (1976) Heterotransplantation of human malignant melanomas to the mouse mutant nude. *Acta Pathol. Microbiol. Scand.*, **9**, 16.

REEVES, B. R. & HOUGHTON, J. A. (1978) Serial cytogenetic studies of human colonic tumour xenografts. *Br. J. Cancer*, **37**, 612.

RYGAARD, J. & POVLSEN, C. O. (1969) Heterotransplantation of a human malignant tumour to 'nude' mice. *Acta Pathol. Microbiol. Scand.*, **77**, 758.

SECOND INTERNATIONAL WORKSHOP ON NUDE MICE (1977) *Proceedings*, Ed. Nomura *et al.* Stuttgart: Fischer Verlag.

SELBY, P. J., HEYDERMAN, E., GIBBS, J., PECKHAM, M. J. (1979) A human testicular teratoma serially transplanted in immune-deprived mice. *Br. J. Cancer*, **39**, 578.

SHARKEY, F. E., FOGH, J. M., HAJDU, S. I., FITZGERALD, P. J. & FOGH, J. (1977) Experience with heterotransplanted human tumours in the nude mouse. *Am. J. Pathol.*, **86**, 29.

SHIMOSATO, Y., KAMEYA, T., NAGAI, K. & 4 others (1976) Transplantation of human tumours in nude mice. *J. Natl Cancer Inst.*, **56**, 1251.

STEEL, G. G. (1977) *Growth Kinetics of Tumours.* Oxford: Clarendon Press.

STEEL, G. G., COURTENAY, V. D. & ROSTOM, A. Y. (1978) Improved immune-suppression techniques for the xenografting of human tumours. *Br. J. Cancer*, **37**, 224.

TAKAHASHI, S., KAKATANI, K., SHIRATORI, T., KONISHI, Y. & KOJIMA, K. (1977) Heterotransplantation of the human stomach carcinomas into nude mice. *Jap. J. Gastroenterol.*, **74**, 431.